# Surgical Treatment of Dyskinesia in Parkinson’s Disease

**DOI:** 10.3389/fneur.2014.00065

**Published:** 2014-04-29

**Authors:** Renato P. Munhoz, Antonio Cerasa, Michael S. Okun

**Affiliations:** ^1^Division of Neurology, Department of Medicine, University of Toronto, Toronto Western Hospital, Toronto, ON, Canada; ^2^Neuroimaging Unit, Institute of Molecular Bioimaging and Physiology, National Research Council (IBFM-CNR), Germaneto, Italy; ^3^Magna Græcia University of Catanzaro, Germaneto, Italy; ^4^Department of Neurology, McKnight Brain Institute, University of Florida College of Medicine, Gainesville, FL, USA

**Keywords:** Parkinson’s disease, dyskinesia, deep brain stimulation, DBS, pallidotomy

## Abstract

One of the main indications for stereotactic surgery in Parkinson’s disease (PD) is the control of levodopa-induced dyskinesia. This can be achieved by pallidotomy and globus pallidus internus (GPi) deep brain stimulation (DBS) or by subthalamotomy and subthalamic nucleus (STN) DBS, which usually allow for a cut down in the dosage of levodopa. DBS has assumed a pivotal role in stereotactic surgical treatment of PD and, in fact, ablative procedures are currently considered surrogates, particularly when bilateral procedures are required, as DBS does not produce a brain lesion and the stimulator can be programed to induce better therapeutic effects while minimizing adverse effects. Interventions in either the STN and the GPi seem to be similar in controlling most of the other motor aspects of PD, nonetheless, GPi surgery seems to induce a more particular and direct effect on dyskinesia, while the anti-dyskinetic effect of STN interventions is mostly dependent on a reduction of dopaminergic drug dosages. Hence, the *si ne qua non-condition* for a reduction of dyskinesia when STN interventions are intended is their ability to allow for a reduction of levodopa dosage. Pallidal surgery is indicated when dyskinesia is a dose-limiting factor for maintaining or introducing higher adequate levels of dopaminergic therapy. Also medications used for the treatment of PD may be useful for the improvement of several non-motor aspects of the disease, including sleep, psychiatric, and cognitive domains, therefore, dose reduction of medication withdrawal are not always a fruitful objective.

## Introduction

Treatment of levodopa-induced dyskinesia (LID) is one of the most common indications for stereotactic surgery in Parkinson’s disease (PD). Control of LID can be accomplished by providing significant relief on the motor symptoms of PD through medication optimization, typically through subthalamic nucleus (STN) deep brain stimulation (DBS), or by pallidotomy or globus pallidus internus (GPi) DBS, which are thought to have a direct effect on dyskinesia (1). Currently, DBS has become the preferred stereotactic procedure in PD, however, ablative surgery continues to be performed and can be quite effective especially when dyskinesia are significantly more prominent on one body side. In this review, we will address both forms of surgical techniques, their indications, differentials, and outcomes.

## Ablative Surgery

The indication for any form of stereotactic ablative surgery has always been the symptomatic treatment of certain motor features of PD as identified during a detailed multi-disciplinary workup. As early as the 50s, the inner segment of the GPi and the ansa lenticularis has been the common choice for functional neurosurgeons (2). This approach was advocated and further reinforced following the observation that ligation of the anterior choroidal artery, performed for the treatment of accidental bleeding in a PD patient, resulted in relief of tremor (3, 4). As this technique (ansa – pallidotomy) became more widely utilized, the results for tremor control were mixed, despite the good outcome for rigidity. As a result, pallidotomy was gradually replaced by thalamotomy, which exerted a more optimal tremor control (5). The failure to reduce tremor in some cases was likely due to a failure to target the appropriate postero-ventral pallidum, which was a limitation of early technology. However, with the advent of levodopa in the 1960s, stereotactic surgery became gradually less popular and did not re-emerge again until the early 90s (6).

The renaissance of stereotactic surgery for patients with PD was galvanized by the weaknesses of levodopa therapy that were gradually surfacing (7). The first disappointment was the evidence that it does not interfere positively with disease progression, as it is now well appreciated after decades of use. Additionally, due to the high doses sometimes required to improve tremor, motor fluctuations, and LID started to be noticed. As most clinicians continue to witness today, medical pharmacological management of PD is challenging with the continuous attempt to balance the relief of parkinsonian motor signs against motor fluctuations and induction of dyskinesia, often with neither being managed adequately (8). The setback related to levodopa was allied with advancements from physiological and surgical aspects, and also by the more accurate understanding of the organization of the basal ganglia (BG), better surgical techniques, and the use of neuroimaging for more precise target localization. At that time, thalamotomy was revived, and, in addition to improvements in the motor aspects of PD, several authors reported drastic suppression of LID (9).

Svennilson et al. reported that when the postero-ventral GPi was lesioned additional benefit to general motor function (interpreted as corresponding to relief of akinesia) could be obtained (10). Later, Laitinen et al. returned to the initial target and the postero-ventral GPi became the preferred surgical treatment for PD patients. In the classical report from 1992, Laitinen’s group showed favorable outcomes that included not only robust improvements in the cardinal signs of PD, but also significant amelioration of LID (11).

At approximately the same time, anatomic and physiological studies confirmed that the GPi and STN were both overactive in PD. Also, experimental studies demonstrated that lesions in these structures could improve parkinsonism and dyskinesia in animal models (12–14), although the classical BG model predicted that pallidotomy would worsen dyskinesia. This discrepancy between theoretical and practical outcomes is probably the result of interference with abnormal firing patterns (rather than rates) in circuit neurons (15).

Both the clinical report by Laitinen et al. and the pathophysiological laboratory-based developments in PD reinforced the role of pallidotomy for treatment of LID in PD.

### Pallidotomy

During the second half of the 90s, many studies reported that unilateral pallidotomy in patients with PD provided successful control of contralateral dyskinesia. At that time, Lozano et al. (15) published the results of pallidotomy in 14 patients with rigid akinetic PD, complicated by severe LID, motor fluctuations, but with intact cognition. Motor improvements in the OFF medication condition were mainly contralateral. The most dramatic improvement was for ON period LID, which were shown to be reduced by 92% in the contralateral side after 6 months. The typical complications of this procedure, homonymous hemianopia, facial paresis, and hemiparesis, were not observed, except for mild and transient facial droop in three cases (noted by clinicians but not the patients). Two years later, the same group published a series of 40 cases, some with a longer follow-up: 27 for 1 year and 11 for 2 years. Short term results were similar to their previous study, however trend analysis revealed a slight worsening of contralateral dyskinesia after the first year, and a loss of benefit for ipsilateral dyskinesia by the second year. Age had an impact on OFF period motor signs, with those older than 65 retaining less improvement after 6 months. LID responded similarly in the two age groups studied. There were no significant reductions in dopaminergic therapy after surgery. Persistent adverse events included facial weakness (two cases), bulbar deficits (three cases), mild dementia (three cases), and worsening of handwriting in four cases (16). The findings in the same cohort were also described after a much longer follow-up (mean 52 months), showing a sustained improvement in OFF period contralateral motor signs and in LID. Other than dyskinesia and levodopa responsive motor signs, no additional characteristics had a significant impact on long-term surgical outcome (17).

In 1998, another group published a preliminary study with a series of 26 PD patients, confirming that the most significant effect following unilateral ventral medial pallidotomy was the reduction of contralateral LID by 67%, while ipsilateral and axial dyskinesia also improved (both around 50%) significantly. The improvement in underlying parkinsonism as measured by comparing the Unified Parkinson’s Disease Rating Scale (UPDRS) scores in the OFF state before and 3 months after surgery, was less robust (27%). On medication, no significant post-operative improvements in parkinsonism were detected and antiparkinsonian medication dosages increased by 11% post-operatively. The presence of disabling LID, therefore, was considered the major indication for this surgical procedure. Two (7.7%) patients died due to cerebral hemorrhages directly related to surgery, while another 15% had major complications (significant focal motor and bulbar deficits) (18). In 2003, the first randomized, prospective controlled trial comparing pallidotomy with best medical therapy was published. The study included 18 patients in each group, showing, after 6 months, a 32% improvement of the total UPDRS motor score in the surgical group versus only a 5% deterioration for those randomized for medical therapy. Mean score improvement in the dyskinesia section of the UPDRS (Part IV) for the surgical group was 45%, whereas patients kept on medical therapy worsened by 8%. The study also revealed that LID improved after pallidotomy in all patients, and two-thirds had “complete relief” on the contralateral side. Also, there was a 36% reduction in ipsilateral dyskinesia severity. Levodopa equivalent doses remained unchanged. There were no fatal outcomes and complications occurred in three cases (16.7%). This study also showed that the age had a clear relationship with clinical outcome, independent of disease duration, with younger patients showing more improvement. This effect was continuous, with no apparent threshold (19).

The series with the longest follow-up was by Kleiner-Fisman et al. and included 10 patients, showing a trend toward significance lasting up to 12 years in contralateral LID (20), and by Hariz and Bergenheim who reviewed 13 of the 38 patients described in Laitinen’s original study from 1992. Mean follow-up was 10.5 years (up to 13.5), and the effect of surgery remained consistent for contralateral LID, but varied for the appendicular OFF period signs. The authors went as far as to consider pallidotomy as a prophylactic measure against LID (21).

Lesion size does not seem to have a significant effect on response (22), however, the optimal location within the GPi that improved dyskinesia is a matter of controversy. Lesion location and size may be different from that required to ameliorate other PD signs, and some experts still advocate a bigger lesion size for prolonged benefit. While anteromedially placed lesions seems to be better correlated with improvement in contralateral LID, central and posterolateral placed lesions improved OFF parkinsonian signs (23). However, lesions placed in more ventral locations or anywhere in the postero-ventral GPi have been shown to be equally effective (16). Differences in outcome measures as well as in methods of determining lesion location probably account for many of these discrepancies.

Bilateral pallidotomies, staged and simultaneous, produce similar improvements in OFF motor and ON dyskinesia to unilateral procedures, with the possible advantage of improvements in axial dyskinesia, dystonia, and, arguably, selected aspects of gait such as walking speed and freezing (24, 25). These good results were undermined by unacceptable cognitive and bulbar (mainly speech) adverse effects (26). Only a few other studies have shown different perspectives (27, 28). The series by Parkin et al. (29) for instance, showed the results in 115 patients who underwent pallidotomy, 53 of which consisted of bilateral procedures. These authors reported significant effectiveness for bilateral pallidotomy, especially for dyskinesia for up to 12 months, at the expense of worsening of speech in 8% and salivation in 13%, figures that were similar to those found for unilateral surgery. Table 1 shows a summary of the advantages, disadvantages, and other aspects to be considered when a pallidotomy is indicated.

**Table 1:** **Patient selection and point to be considered when indicating stereotactic surgery for dyskinesias in Parkinson’s disease**.

	Advantages	Disadvantages	Patient profile	Post-operative details
**Unilateral pallidotomy**	Efficacious	Permanent lesion	Unable to travel	Ipsilateral dyskinesias may not improve significantly, requiring continuing anti-dyskinetic medical treatment or contralateral GPi DBS
	Less costly than DBS	Not reversible	Live where DBS is too expensive or not available Prefer not to have chronic hardware	
	Does not require post-operative programming	Bilateral surgery has higher risk of side effects		
	No complications related to hardware (infections, malfunction)	Does not allow adjustments to control side effects	High infection risk	
**GPi DBS**	Direct improvement in dyskinesias Allows *adjustments* in drug regimen May allow for maintaining or introducing higher levels of dopaminergic therapy Synergistic effect with l-DOPA on axial and other symptoms	No significant change in drug regimen in many but not all cases Ventral and dorsal stimulation may induce opposite effects on cardinal motor signs of PD however this has not been replicable on all cases	Needs prompt improvement of severe dyskinesias	Ensure that the beneficial effect of l-DOPA is not antagonized by stimulation
			Responds to low dose l-DOPA, but has low threshold for dyskinesias	
			Has l-DOPA responsive non-motor signs In the right context, may be safer for patients with mild pre-existing cognitive symptoms	
**STN DBS**	Allows significant *reduction* in dopaminergic drug dosages Effective for OFF period dystonia	Improvements in dyskinesias depend on reduction of levodopa May have negative impact on cognition	Has severe motor fluctuations Uses higher doses of l-DOPA Experiences disabling side effect of dopaminergic treatment Intact cognition	Stimulation induced dyskinesias may appear after a latency of several hours if l-DOPA not adjusted The electrode that induces dyskinesias is usually the most effective
		More laborious post-operative management		
		May worsen or not improve dyskinesia in brittle dyskinetics		

### Studies comparing pallidotomy and deep brain stimulation of the GPi or STN

Few studies have compared the efficacy and safety of pallidotomy and DBS of the GPi or STN. Table 2 shows the results of the most important clinical parameters described in studies of pallidotomy and DBS techniques. An early, non-randomized trial comparing results of pallidotomy, STN, and GPi DBS concluded that GPi DBS had effects similar effects to pallidotomy, but is safer when bilateral procedures are required. Also, bilateral STN DBS may improve OFF period motor symptoms to a greater proportion than the other procedures, and might also improve ON period motor function (30). In 2004, Esselink et al. (31) compared, in a randomized, observer-blind trial, the effect of unilateral pallidotomy and bilateral STN DBS in patients with PD followed up for 6 months, confirming that stimulation was more effective in reducing OFF period motor signs. In addition, this procedure provided better ON period motor scores and a greater reduction of dopaminergic drug treatment dosages. Both improved LID and functional scales equally, and the number of adverse events was similar in both groups. The same group also published the results after 4 years with similar findings, except for dopaminergic treatment dosage, which did not significantly differ between groups after the first 12 months (32).

**Table 2:** **Effects of unilateral pallidotomy, bilateral GPi and STN deep brain stimulation (DBS) on general motor improvement (UPDRS III), dyskinesias (UPDRS IV) and levodopa equivalent daily dose (LEDD)**.

	Motor improvement (%)	Improvement for dyskinesias (%)	Reduction in LEDD
**Unilateral pallidotomy**	25–45	45–86	n.s. (0–10%)
**GPi DBS**	26–43	47–88	n.s. (15–17%)
**STN DBS**	25–54	20–83	31–47%

*Mean improvement after a minimum of 6 months compared to preoperative baseline. Scores reflect the medication off condition; for DBS, stimulation on. n.s., non-significant (17, 32–41)*.

## Deep Brain Stimulation

After DBS was introduced as a treatment option for movement disorders in general, this technique has slowly taken over a pivotal central role in stereotactic surgery. As a matter of fact, ablative procedures are currently regarded as alternatives, only used when DBS is not viable due to technical, travel, patient preference, and economic reasons (42, 43).

Two of the reasons that favor DBS, particularly if bilateral procedures are required, are the facts that it is not intended to produce a brain lesion, and that the stimulator can be programed with respect to several variables, including electrode location, amplitude, frequency, and pulse width, to induce better therapeutic effects while minimizing adverse effects. In the case of PD, DBS electrodes have been placed in two main BG targets: the GPi, and the STN, though other targets are also possible (44).

### GPi DBS

The first study to report results of this procedure described three patients in 1994, with the post-operative results described as “excellent,” reflecting improvements in all motor signs of the disease, as well as for motor fluctuations and LID (45). During the following decade, descriptions of larger series confirmed these findings. A study with a follow-up of at least 24 months showed that the mean improvements in the UPDRS motor and activities of daily living scores after 12 months were more than 50%, motor fluctuations were reduced from 40 to 10%, and the score for LID was reduced to one third. Doses of levodopa tended to remain unchanged. Half of the patients experienced a slight worsening of levodopa and stimulation resistant gait and bulbar symptoms following 12 months (46). In 2000, a study by Kumar et al. (34) showed the results seen on a cohort of 22 consecutive cases of PD treated with GPi DBS, 17 of whom had bilateral surgeries. Post-operatively, at 6 months, the motor improvement in the OFF condition reached 31 and 66% reduction in LID.

The first double-blind, crossover study evaluating the results of GPi and STN DBS in PD was performed in 2001, showing that both procedures induced significant improvements in motor function and dyskinesia (by 58% for STN and 66% for GPi DBS), however, the average medication used, measured in levodopa equivalents, decreased significantly more for the STN DBS patients (35). A study with longer follow-up, mean 48.5 months, showed a 64% mean improvement in dyskinesia after this period (36). Finally, another study followed up 11 patients with PD who underwent GPi DBS for up to 5 years, showing that, despite a decline on the motor benefit for the OFF period scores after 3 years, the improvement in LID was sustained for up to 5 years (47).

### STN DBS

STN DBS for advanced PD was first introduced in the 1990s and is currently the most common form of a surgical treatment applied for this disorder worldwide. The initial series reported significant improvements in OFF period tremor, rigidity, and bradykinesia, as well as attenuation of motor fluctuations and LID, associated with a 50% reduction in dopaminergic treatment dosages (48). Subsequent studies confirmed these findings. In 2001, a prospective study of 91 patients showed, after 6 months, a robust improvement in all motor signs in the OFF condition, in the percentage of time with good mobility and no dyskinesia, mean dyskinesia score, as well as a mean reduction in daily levodopa dose equivalents (approximately 60%) (35). At this point, it became clear that the reduction in dyskinesia could be attributed at least in part to the reduction of levodopa dosage. However, a few studies showed that this may not be the only element in this beneficial effect. A study designed to assess the effect of STN DBS on OFF period dystonia, and on diphasic and peak dose dyskinesia after a levodopa challenge using the same suprathreshold dose as before surgery with the stimulation on, showed a reduction of OFF period dystonia by 90%, and of diphasic dyskinesia by 50%, and of peak dose dyskinesia by 30% (49). The same authors had already reported that chronic STN DBS *per se* tends to reduce dyskinesia, as opposed to chronic activation of the dopaminergic system with levodopa. The authors speculated that this difference may have been due to the pulsatile nature of levodopa stimulation *versus* the more continuous activation provided by chronic STN DBS (50). There was also an important study by Oyama et al. that elegantly showed that dyskinesia could possibly be reduced in both the STN and GPi target. The authors accounted for medication reduction, and showed that in both targets there was a possibility of dyskinesia suppression without medication reduction (51).

Long-term studies of bilateral STN DBS in patients with advanced PD have demonstrated the stability of this therapy over time. A 5-year prospective study of 49 consecutive patients treated with STN DBS noted that OFF medication motor scores at 5 years were still 54% better than baseline (37). Worsening of ON medication akinesia, speech, postural stability, and freezing of gait was interpreted to be consistent with the natural progression of PD. However, LID benefits persisted, with dyskinesia disability and duration at 5 years being improved by 58 and 71%, respectively in comparison with baseline. Similar benefits with respect to dyskinesia were observed in 37 patients followed for 5 years after DBS surgery (52). Finally, a comprehensive meta-analysis of 921 patients who underwent STN DBS between 1993 and 2004 noted an average reduction in LID of 69.1% (53).

### Mechanisms of action in reducing levodopa-induced dyskinesia

#### Pallidal stimulation

Restoration of the thalamocortical activity by suppression of the inhibitory output from the pallidum to the ventrolateral thalamus is the suspected mechanism for motor improvement underpinning GPi DBS, however, the cellular mechanisms of high-frequency stimulation are still unknown. The mechanism of GPi DBS in reducing dyskinesia is also not completely understood. The current views of the BG physiology suggest that inhibition of ventral GPi activity should induce dyskinesia, however, lesioning of the ventral pallidum provides relief of dyskinesia (54). One of the possible justifications for this apparent paradoxical response is that LID may be more correlated with an abnormal pattern than with the direction and intensity of the neuronal activity within the GPi (54, 55). Surgical modification of this patterned activity might be accomplished by lesioning (direct neuronal inhibition) or with DBS (indirect inhibition through activation of inhibitory axons close to the electrode). Dyskinesia might also arise from an abnormal balance of activity within different functional zones of the nucleus (ventral versus dorsal GPi) and stimulation may suppress this abnormal activity (56, 57). Finally, the anti-dyskinetic effect of GPi DBS maybe mediated through effects on the subthalamopallidal tract, which projects to the dorsal GP externus and GPi. Dorsal GPi stimulation might inhibit this projection and would be expected to improve PD symptoms and induce dyskinesia (58).

#### STN stimulation

STN DBS mimics the effects of levodopa on parkinsonian motor symptoms and allows reduction of dopaminergic medication, secondarily relieving dyskinesia as medications are reduced or withdrawn post-operatively (51). However, improvement of dyskinesia is also sometimes observed in the early post-operative period after implantation of electrodes in the STN, even in the absence of a reduction of medications (1). This indicates a direct anti-dyskinetic effect of manipulation of the STN (or the vicinity of its dorsal border and perhaps the zona incerta), but long-term relief of dyskinesia generally requires reduction of medications. The specific site of action in stimulation of the STN is unknown. Some data indicate that the best effect can be achieved at the lowest intensity not through stimulation of neurons within the STN, but by stimulation of tissue dorsal to it, which might affect the pallidothalamic bundle, the pallidosubthalamic tract, and/or the zona incerta (59). Other data indicate that the most effective contact location appears to be within the anterodorsal portion of the STN, although current could spread from this location into the directly superior fields of Forel and zona incerta (60). The observation that an active DBS contact dorsal to the STN may provide better control of dyskinesia (indicative of a direct anti-dyskinetic effect) supports the notion that activation of structures dorsal to the STN is important in providing relief of parkinsonian symptoms by DBS of the STN (38). Overall, the specific mechanisms of action of GPi and STN DBS in suppressing dyskinesia are unknown.

### Studies comparing the effect of GPi and STN DBS

A few studies have compared the effect of GPi and STN DBS on PD. Table 2 shows the results of the most important clinical parameters described in studies comparing these two techniques. The first study to do that was published in 2001, it had a relatively short follow-up period after surgery (3 months), and revealed similar improvements in OFF period motor parameters, as well as for ON dyskinesia, with the caveat that only the STN group was able to significantly reduce the levodopa equivalent dose (35). In 2005, a non-randomized extension of this study with 105 patients followed up for at least 3 years, showed that, in addition to improvements in all motor signs of parkinsonism in the OFF condition, STN DBS significantly improved OFF dystonia and ON dyskinesia, while GPi had a similar effect on ON dyskinesia with no significant improvement on OFF dystonia. In this study, reduction in post-operative levodopa equivalent doses was significant only for the STN group, in which more than 10% of patients stopped taking levodopa. These changes were sustained after up to 4 years of follow-up (36). Moro et al., in an double-blind, non-randomized study with 35 patients who underwent STN DBS and 16 who underwent GPi DBS, found that both procedures induced significant improvements in OFF period motor signs, ADLs, and ON dyskinesia scores, although only the STN group had a significant reduction in the doses of levodopa. These results were sustained after 6 years of follow-up (61). A direct comparison of both procedures was published in 2012 (39). This was a randomized, evaluator-blind study with 198 PD patients followed up for at least 36 months, which concluded that the primary outcome, OFF period motor improvement (including subscales for each motor sign), was significantly improved, but the improvements were similar, stable over time, and with parallel trends for both targets. The scores for complications of levodopa therapy (UPDRS IV), including dyskinesia, as well as the amount of ON period time without troublesome LID were significantly improved for both groups over 36 months, with non-significant, but greater decreases in levodopa dosages in the STN group. Finally, one recent double-blind study of 128 PD patients randomized for either form of treatment, showed that patients who underwent STN DBS had larger improvements in OFF period mean UPDRS motor score, mean change in ADLs scores and mean reduction in medication after surgery. OFF dystonia scores were similarly improved as well as the time in ON phase without dyskinesia. The scores of the dyskinesia rating scales were significantly better 12 months after surgery for those who underwent GPi DBS. This difference probably occurred because the authors assessed patients after 12 months with the same dose of levodopa used at baseline, however in daily life, they may use lower doses, leading to less LID (40).

### Practical issues; selection of the surgical target, technique, and programing

Table 1 shows a summary of points that need to be considered when indicating these DBS techniques.

Numerous studies have demonstrated the effectiveness of STN DBS in controlling the appendicular motor signs of PD, however, this procedure is not considered to have as much of a direct effect on the intensity of LID. The anti-dyskinetic effect of STN DBS have been hypothesized to be related to allowing reduction of dopaminergic drug dosages, with consequent improvement in side effects, including LID. The persistence or worsening of LID after STN DBS is common and is, in fact, indicative of the necessity to reduce the dose of levodopa (62). Therefore, the *si ne qua non-condition* for reduction of LID when STN DBS is considered, is its capacity to enable a reduction of levodopa dosage. If, however, an adequate response of motor symptoms does not occur post-operatively, dyskinesia will remain unchanged. Of importance, STN stimulation not uncommonly induces contralateral dyskinesia, which may be persistent, and in some cases lead to the implantation of rescue GPi leads.

On the other hand, GPi DBS seems to have a direct effect on the reduction of dyskinesia. Patients undergoing this procedure typically cannot tolerate significantly lower doses of levodopa after surgery, and still appreciate a marked reduction of dyskinesia. Simplistically, patients who experience a good response of their PD symptoms with levodopa, but whose primary and most significant source of disability are dyskinesia may benefit from GPi DBS (51). In other words, GPi DBS can be especially valuable for cases in which LID are a dose-limiting factor for either maintaining or introducing higher but necessary levels of dopaminergic therapy. In addition, levodopa may have a synergistic effect on GPi DBS, which is not seen after STN stimulation. Burchiel et al., for instance, in a randomized, double-blind study, comparing the effects of STN and GPi DBS, showed that, in combination with levodopa, UPDRS motor scores were significantly more improved for patients who underwent the pallidal procedure. This combination was also more clinically significant for axial symptoms, which are traditionally considered refractory to either form of treatment alone (63). Another more recent meta-regression of long-term studies of cases who underwent these procedures confirmed that GPi DBS in combination with levodopa was correlated with better scores for postural instability and gait disorder than STN stimulation plus levodopa (64).

Selection of either target may also be influenced by the fact that medications used for the treatment of PD are useful not only for motor, but also for psychiatric, cognitive, sleep, and other non-motor aspects of the disease, therefore, withdrawal or dose reduction may not be a desired goal (65). Selection of the target should be based on the patient’s most disabling symptoms, response, and side effects related to levodopa, and the ultimate goals of therapy (66). If LID are a patient’s most disabling symptom, especially if they require more immediate improvement due to its severity and potential morbidity, then GPi DBS should be considered with the knowledge that regardless of changes in medication therapy after surgery there is a high likelihood that dyskinesia will improve. On the other hand, patients undergoing STN DBS must hope for a sufficiently good response after surgery that will allow medications to be sufficiently reduced. If change in parkinsonian motor symptoms after STN DBS are insufficient to guarantee reduction of levodopa dosage, or if its reduction worsen or induces non-motor symptoms, the intervention for dyskinesia may be “unfruitful” (1).

In the case of a patient in whom, in addition to motor signs of parkinsonism, medication side effects other than dyskinesia are a primary source of disability (i.e., psychosis, behavioral changes, etc.), STN DBS may be more desirable.

In general, when the presence of LID is the main problem and indication for surgery, there are no formal differences in the procedures when compared to situations when the chief complaint is another motor feature of the disease. However, a few minor variables exist. Implantation of leads is typically performed while patients are in the OFF condition to avoid disabling dyskinesia, leading to motion artifacts during pre operative imaging and to better microelectrode recording during the intraoperative procedure (67). Other variations are used because of possible differential anti-dyskinetic effects of stimulation at different sites within the GPi as stimulation of two different sites within the nucleus induce different effects on dyskinesia and response to dopaminergic treatment. Studies have shown that stimulation of the most dorsal aspects of the GPi in the OFF period usually leads to improvement of the cardinal signs, especially bradykinesia, while inducing dyskinesia, mimicking the action of levodopa. When deeper (ventral) sites within the nucleus were stimulated, signs worsened. In the ON period, stimulation of the ventral GPi reduced dyskinesia but may have worsened bradykinesia. Stimulation of the intermediate area seems to provide a balance between these two extremes. It is unclear whether these findings have a practical significance, but their existence should be kept in mind during surgical planning, positioning of the lead within the GPi, and during programing sessions (56, 57).

#### Post-operative programing: GPi DBS

As a rule, the evaluation of stimulation-related beneficial effects is typically less reliable during the first weeks after electrode implantation, due to the lesion effect of the procedure. Therefore, the initial programing should be performed after at least 2 weeks of surgery. At this time, the patient should be in the OFF medication condition, after 12 h of dopaminergic drug withdrawal. The first step should focus on achieving the best improvement of the cardinal signs of parkinsonism. The second phase should address the patient during the ON period, under the effect of levodopa, with particular awareness for LID. Therefore, the goal of programing should be attempting to achieve a good relief of PD symptoms in the OFF condition, not associated with the occurrence of dyskinesia in the ON period, and with the highest threshold for side effects of stimulation. This procedure should be performed for all four contacts separately, defining a hierarchy for therapeutic window (68).

In patients whose primary complaint is LID, an additional programing session can be performed in a full ON condition to confirm the adequate beneficial effect of stimulation, but is usually only indicated if there are difficulties suppressing dyskinesia. Special attention must be directed to ensure that beneficial medication effects are not antagonized by stimulation, as well as the OFF medication symptoms are not exacerbated, since different regions within the GPi may have opposite effects on dyskinesia and on the cardinal signs of parkinsonism, when stimulated. Fortunately, the detrimental effects of stimulation on parkinsonism and the response to levodopa have higher thresholds than the beneficial effects on dyskinesia. As ventral GPi areas may provide good relief of dyskinesia at the expense of loss of beneficial effect of levodopa, a better stimulation response can be detected by using more central contacts, which usually provide good relief of dyskinesia as well as tremor, rigidity, and bradykinesia (56, 69). It is important to point out that many experts have been unable to replicate the differential effects of programing different contacts in the GPi, and that in general the GPi has been found to be a much easier target to optimize. The GPi target also allows for more flexibility that the STN target, as was recently shown by Weaver et al. in VA study 36 months outcomes (39, 70).

#### Post-operative programing: STN DBS

As STN DBS ideally mimics the motor effects of levodopa in many aspects, the main objective of initial programing in cases of dyskinesia relies on providing a significant improvement of the motor signs of parkinsonism and a concomitant decrease in levodopa dosage, which, on average, reaches a 50% reduction (41). Therefore, as in the case of GPi DBS, the first programing session should preferentially be performed in patients during the OFF period, holding all medications for PD for 12 h. In fact, most experts that program STN and GPi DBS have patients report to the clinic in an OFF medication condition, which provides a nearly optimal programing scenario (no bias of medications). This is generally enough for most patients, however some patients may require longer OFF periods. In difficult cases, after programing for reduction of bradykinesia, tremor, and, especially, rigidity, patients should take their regular doses of levodopa and, in the ON state, be assessed for adverse effects with the combination of stimulation and medications, particularly dyskinesia. The patient should be seen during this first session at the peak effect of levodopa, and ideally should have access to expert programing for the next few days, as dyskinesia may appear after a latency period of up to several hours (71, 72). During the first few weeks and months after surgery, as stimulation is adjusted to provide the best relief of parkinsonian symptoms, medication doses can be slowly titrated downward, and LID tends to improve or resolve. Moreover, dyskinesia has been hypothesized to improve with chronic continuous stimulation due to plastic changes as a direct effect of stimulation, leading to desensitization of the neuronal circuitry underlying to LID. Persistent dyskinesia is generally treated by further reduction of medication (71).

In some instances, especially during the first few weeks after DBS implantation, dyskinesia may be exacerbated and, in fact, the induction of these involuntary movements in the short term predicts a favorable long-term outcome (51). Thus, the particular electrode that induces dyskinesia is usually the most effective contact for long-term therapy. In these cases, if reducing levodopa leads to worsening of PD symptoms, medication doses should be kept at the lowest adequate therapeutic level, and stimulation amplitudes or other parameters should be reduced. Over time, the threshold for induction of dyskinesia typically increases, and amplitude can be gradually increased (73). Finally, if stimulation using the most effective contact precipitates dyskinesia that cannot be controlled except by unacceptable reduction of stimulation intensity, programing the system to use a more proximal contact in a monopolar configuration, or reprograming to a bipolar configuration may be necessary. Addition of a contact dorsal to the STN (perhaps in the zona incerta) may also provide better control of dyskinesia (71).

## Conclusion

Although STN and GPi procedures have different mechanisms of action, both are effective treatments strategies to control LID. GPi interventions may have a more immediate effect, independent of reduction of levodopa daily dosage. On the other hand, several centers tend to adopt STN DBS as this procedure also brings marginally better improvements in OFF period motor scores than GPI DBS, as indicated by a recent randomized controlled trial (40). Overall, selection of the surgical target should be based on each patient’s most disabling symptoms, medication response and regimen, and goals of therapeutic intervention. Currently, the literature is almost entirely focused on results that analyze a combination of the best possible results in regards to global improvements, therefore the ideal stimulation parameters specific for the control of LID are unknown. Also, the anti-dyskinetic effects of additional or combined targets, such as external and internal pallidal stimulation, and the use of adaptive DBS remain largely unexplored.

## Conflict of Interest Statement

The authors declare that the research was conducted in the absence of any commercial or financial relationships that could be construed as a potential conflict of interest.
